# Revefenacin, a Long‐Acting Muscarinic Antagonist, Does Not Prolong QT Interval in Healthy Subjects: Results of a Placebo‐ and Positive‐Controlled Thorough QT Study

**DOI:** 10.1002/cpdd.732

**Published:** 2019-08-29

**Authors:** Marie T. Borin, Chris N. Barnes, Borje Darpo, Srikanth Pendyala, Hongqi Xue, David L. Bourdet

**Affiliations:** ^1^ Theravance Biopharma US, Inc. South San Francisco California USA; ^2^ ERT, previously iCardiac Technologies Rochester New York USA

**Keywords:** COPD, healthy subjects, long‐acting muscarinic antagonist, moxifloxacin, nebulized, revefenacin

## Abstract

Revefenacin is a novel once‐daily, lung‐selective, long‐acting muscarinic antagonist developed as a nebulized inhalation solution for the maintenance treatment of chronic obstructive pulmonary disease. In a randomized, 4‐way crossover study, healthy subjects received a single inhaled dose of revefenacin 175 µg (therapeutic dose), revefenacin 700 µg (supratherapeutic dose), and placebo via standard jet nebulizer, and a single oral dose of moxifloxacin 400 mg (open‐label) in separate treatment periods. Electrocardiograms were recorded, and pharmacokinetic samples were collected serially after dosing. The primary end point was the placebo‐corrected change from baseline QT interval corrected for heart rate using Fridericia's formula, analyzed at each postdose time. Concentration‐QTc modeling was also performed. Following administration of revefenacin 175  and 700 µg, placebo‐corrected change from baseline QTcF (ΔΔQTcF) values were close to 0 at all times, with the largest mean ΔΔQTcF of 1.0 millisecond (95% confidence interval [CI], −1.2 to 3.1 milliseconds) 8 hours postdose and 1.0 millisecond (95%CI, −1.1 to 3.1 milliseconds) 1 hour postdose after inhalation of revefenacin 175 and 700 µg, respectively. Revefenacin did not have a clinically meaningful effect on heart rate (within ±5 beats per minute of placebo), or PR and QRS intervals (within ±3 and ±1 milliseconds of placebo, respectively). Using concentration‐QTc modeling, an effect of revefenacin > 10 milliseconds can be excluded within the observed plasma concentration range of up to ≈3 ng/mL. Both doses of revefenacin were well tolerated. These results demonstrate that revefenacin does not prolong the QT interval.

Cardiovascular disease, including cardiac arrhythmias, represents one of the most common comorbidities in the growing population of people with chronic obstructive pulmonary disease (COPD).^1^ Revefenacin (YUPELRI®) inhalation solution, delivered via standard jet nebulization, is an anticholinergic indicated for the maintenance treatment of patients with chronic obstructive pulmonary disease (COPD).^2^, ^3^, ^4^ After inhaled administration, revefenacin is rapidly absorbed, with peak concentrations observed within 14 to 31 minutes after the start of nebulization. Absolute bioavailability after oral administration is low (<3%).^4^ The primary metabolic pathway for revefenacin is via hydrolysis to form THRX‐195518,^5^ with only a minor contribution of cytochrome P450 enzymes. Results of binding studies at human recombinant M3 (hM3) muscarinic receptors show that THRX‐195518 possesses modest muscarinic antagonistic activity but is 10‐fold less potent than revefenacin and dissociates more rapidly from hM3 receptors. Revefenacin is not anticipated to be subject to drug‐drug interactions; however, its major metabolite (THRX‐195518) is a substrate of organic anion‐transporting polypeptide 1B1 (OATP1B1) and OATP1B3, and therefore coadministration of revefenacin with OATP inhibitors could lead to increased metabolite exposure.^4^ Limited plasma accumulation (<1.6‐fold) is observed for both revefenacin and THRX‐195518 after multiple‐dose (daily) inhaled administration.^4^

This novel once‐daily, lung‐selective, long‐acting muscarinic antagonist (LAMA), is predicted to have a low potential for adverse cardiovascular effects, including QT prolongation, as it does not inhibit the human ether‐à‐go‐go (hERG)–related gene potassium channel at concentrations well above those observed clinically. In HEK293 cells stably transfected with hERG cDNA, the hERG half‐maximal inhibitory concentrations (IC_50_) for revefenacin and THRX‐195518 were 43 000‐fold and 614 000‐fold higher, respectively, than the maximum unbound plasma concentration (C_max_) values in subjects receiving 700 µg of revefenacin (data on file, Theravance Biopharma US, Inc.).

Although no clinically meaningful changes in electrocardiogram (ECG) recordings were observed with revefenacin in previous studies,^6^, ^7^ clinical data on cardiac repolarization are needed to provide a complete assessment of the proarrhythmic risk. Thus, it is important to evaluate robust ECG assessments, including at the highest anticipated plasma levels of the drug, and evaluate the heart rate–corrected QT interval prolongation risk with confidence. Cardiac safety data are presented from a phase 1 randomized, partially double‐blind (except moxifloxacin), single‐dose, 4‐way crossover, placebo‐ and positive‐controlled thorough QT study in healthy subjects. This study was conducted to evaluate the potential for changes in QTc following administration of inhaled revefenacin at therapeutic and supratherapeutic doses.

## Methods

### Study Design

This phase 1 single‐center, randomized, partially double‐blind, placebo‐ and positive‐controlled, single‐dose, 4‐way crossover study (NCT02820311) was conducted at Celerion (Tempe, Arizona) in accordance with the principles of the International Council for Harmonisation (ICH) of Technical Requirements for Registration of Pharmaceuticals for Human Use Guideline for Good Clinical Practice,^8^ the United States Code of Federal Regulations, the principles of the World Medical Association Declaration of Helsinki—Ethical Principles for Medical Research Involving Human Subjects, and all applicable regulatory requirements, including the archiving of essential documents. The study protocol was reviewed and approved by Chesapeake Research Review, Inc. (Columbia, Maryland).

### Study Population

The study population included healthy, nonsmoking male and female subjects, 18 to 55 years of age. Key inclusion criteria were the subject's ability and willingness to provide written informed consent, body mass index of 19 to 32 kg/m^2^, and normal blood pressure and heart rate (HR) measured after resting seated or supine for approximately 5 minutes. Subjects with clinically significant abnormal ECG findings at screening, a known history of cardiovascular disease, a known family history of congenital long QT syndrome, or a known family history of sudden death were excluded from the study.

### Study Treatments

In a randomized sequence, each subject received a single dose of the following 4 treatments in separate periods: blinded revefenacin 175 µg, revefenacin 700 µg, placebo via nebulizer (PARI LC Sprint reusable nebulizer; PARI Respiratory Equipment, Inc., Midlothian, Virginia), and open‐label oral moxifloxacin 400 mg (positive control). Subjects were confined for 2 to 3 days in period 1 and for 2 days in the remaining periods. A washout period of ≥14 days between administrations of the study drug was employed and deemed adequate to ensure no carryover treatment effects between study periods.

### Study Assessments

#### ECG Assessment

ECG measurements were recorded serially after dose administration. At the central ECG laboratory (iCardiac Technologies, Rochester, New York), ECGs were extracted in up to ten 14‐second replicates at the following times on day 1 of each period: 60, 45, and 30 minutes before dose administration and 0.25, 0.5, 1, 2, 3, 4, 6, 8, 12, and 24 hours postdose. Continuous digital ECGs were recorded with subjects in a supine or semirecumbent position (having rested for at least 10 minutes) for the first reading up to and including the 4‐hour point, then resting supine or semirecumbent for at least 10 minutes prior to and 5 minutes after the remaining times. TQT Plus software^9^ was used to extract ECGs from continuous 24‐hour recordings, and ECGs were measured using the high‐precision QT technique^10^ by a technician blinded to treatment, time, and study day. All ECGs were read by the same blinded single reader. All measurements were reviewed by a cardiologist. The primary ECG lead was lead II. If lead II was not analyzable, then the primary lead of analysis was changed to V2 or V5, in that order, for the subject's entire data set. Categorical T‐wave morphology analysis and measurement of the PR and QRS intervals were performed using a semiautomated approach in 3 of the 10 ECG replicates at each point.

The primary ECG end point was the placebo‐corrected change‐from‐baseline QT interval corrected for HR using Fridericia's formula (ΔΔQTcF) unless a substantial HR effect was to be observed. A substantial HR effect was defined as the largest placebo‐corrected change‐from‐baseline HR (ΔΔHR) that exceeds 10 beats per minute. In such a case, additional end points for HR correction were to be calculated (subject‐specific QTc, population‐specific QTc, or individualized QTc) and the primary end point chosen based on a prespecified test of HR dependence. Secondary ECG end points included change‐from‐baseline HR, PR interval, and QRS interval and treatment‐emergent T‐wave abnormalities.

#### Pharmacokinetic Assessments

On day 1 of each study period, blood samples for pharmacokinetic (PK) assessment were collected at the following times: before dosing (within 30 minutes before dosing), 15 and 30 minutes postdose, and 1, 2, 3, 4, 6, 8, 12, and 24 hours postdose to quantify the concentrations of revefenacin and its primary metabolite, THRX‐195518, in plasma. Postdose PK sampling times were relative to the start of study drug inhalation (revefenacin or placebo) or oral dosing (moxifloxacin). Concentrations of revefenacin and THRX‐195518 in plasma were quantified using validated liquid chromatography with tandem mass spectrometry methods, which used solid‐phase extraction (strong cation exchange) with stable isotope‐labeled revefenacin‐d5 and THRX‐195518‐d5 as internal standards for quantifying revefenacin and THRX‐195518, respectively, in plasma. The high‐pressure liquid chromatography column was a Mac‐Mod Halo C18 (2.1 × 50 mm; 2.7‐µm particle size) with an in‐line frit (0.5 µm × 2/3 mm). Mobile‐phase (gradient) solvent was 1000:1 water/1 M ammonium bicarbonate adjusted to pH 9 and methanol. The mass spectrometer (AB Sciex API‐6500) was operated under optimized conditions for detection of each analyte in Turbo Ion Spray, positive ionization, and selected reaction monitoring (SRM). The following SRM transitions were used in data acquisition: revefenacin, m/z 598.3 → m/z 302.1; revefenacin‐d5, m/z 602.3 → m/z 306.1; THRX‐195518, m/z 599.3 → m/z 303.1; and THRX‐195518‐d5, m/z 603.3 → m/z 307.1. The lower limit of quantification for revefenacin and THRX‐195518 was 0.0005 and 0.005 ng/mL, respectively. Quality control intra‐assay accuracy (% relative error) was −2.0% to 10% and −4.4% to 6.0%, and interassay accuracy was 3.2% to 6.0% and −2.6% to 3.3% for plasma revefenacin and THRX‐195518, respectively. Intra‐assay precision (% coefficient of variation) for plasma revefenacin and THRX‐195518 was ≤9.6% and ≤6.2%, respectively, and interassay precision was ≤9.1% and ≤4.4%, respectively.

#### Safety Assessments

All subjects were monitored for safety by assessing adverse events (AEs), clinical laboratory tests, vital signs, physical examinations, and 12‐lead safety ECGs.

### Statistical Analyses

#### Sample Size

A sample size of 36 subjects was calculated to provide at least 95% power to demonstrate that the upper bound of the 1‐sided 95% confidence interval (CI) for the largest time‐matched mean difference between treatment and placebo for QTcF excluded 10 milliseconds. The estimated sample size assumed the following: a true treatment difference of 3 milliseconds, a residual standard deviation (SD) of 5.73 milliseconds, and a subject‐by‐time interaction SD of 5.40 milliseconds. To account for potential dropouts and maintain balanced randomization, a total of 48 subjects were randomized to achieve at least 36 evaluable participants with 4 completed treatment periods.

#### ECG Analysis

The main evaluation of the primary ECG end point was the difference in placebo‐corrected change‐from‐baseline QTc (ΔΔQTc) between revefenacin 700 µg and placebo. Change‐from‐baseline QTcF (ΔQTcF) was evaluated 0.25, 0.5, 1, 2, 3, 4, 6, 8, 12, and 24 hours postdose on day 1 of each period. The average (arithmetic mean) of the measured QTc intervals from 3 ECG points recorded 60, 45, and 30 minutes before dose administration on day 1 in each period was used as the baseline. The primary analysis of ΔQTcF was conducted using a linear mixed‐effects model with the following terms: sequence, period, time (categorical), treatment, sex, predose baseline QTcF, and treatment‐by‐time interaction. All times were used to compute model‐based estimates. Subject nested within sequence was included as a random effect term. An unstructured covariance structure was used in the mixed‐effects model. If the unstructured covariance did not converge, a compound symmetric covariance structure was used. Contrasts were constructed to estimate the time‐matched treatment effects and 2‐sided 95%CIs comparing each dose of revefenacin and placebo.

Assay sensitivity for moxifloxacin was established if the lower bound of the 2‐sided 95%CI was greater than 5 milliseconds for the time‐matched mean difference between moxifloxacin and placebo for ΔΔQTc for 1 or more times 1, 2, and 3 hours postdose, using the same model as the analysis for the primary end point. To adjust for multiplicity in the assay sensitivity analysis, a resampling‐based multiple test was carried out. This test accounted for the correlation among the test statistics associated with the moxifloxacin‐placebo comparisons at the postdose times. Central tendency and outlier analyses were performed for both the primary QTc and secondary ECG end points: HR, PR interval, and QRS interval. Categorical outlier analyses were performed to determine the number and percentages of subjects who met specific thresholds for observed and change from baseline values for QTc, HR, PR interval, and QRS interval.

#### Pharmacokinetic Analysis

The following plasma PK parameters for revefenacin and THRX‐195518 were calculated by noncompartmental methods using WinNonlin® Version 6.3: AUC_0‐24_ (area under the plasma concentration‐time curve from time zero to 24 hours postdose), AUC_last_ (area under the plasma concentration time curve from time zero to the time of the last observed/measured nonzero concentration), C_max_ (maximum observed plasma concentration), and T_max_ (time to reach C_max_).

#### Concentration‐QTc Analysis

The relationship between placebo‐corrected change from baseline QTcF (ΔΔQTcF) and plasma concentrations of revefenacin was characterized using a linear mixed‐effect modeling approach of the form ΔΔQTcF*_ij_* = α*_i_* + β*_i_*(Conc*_ij_*) + ε*_ij_*, in which ΔΔQTcF_ij_ was the time‐matched ΔΔQTcF for subject i at time *j* with concentration Conc*_ij_*. The errors, ε_ij_, were assumed to be identical, independent, and normally distributed, with mean 0 and variance σ^2^. Interindividual variability in the exposure‐response parameters (slope and intercept) was modeled using an additive error model Θ*_i_ = TV*Θ* + η*Θ*_i_*, in which Θ*_i_* was the parameter estimate for the *i* ^th^ individual, *TV*Θ was the typical value of the parameter in the population, and *η*Θ*_i_* were individual‐specific random effects for the *i* ^th^ individual and assumed to be normally distributed with mean 0 and variance ω^2^. Furthermore, the following 3 linear models were considered: model 1 was a linear model with a fixed intercept, model 2 was a linear model with a mean intercept fixed to 0 (with variability), and model 3 was a linear model with no intercept. Time‐matched revefenacin concentration was included in the model as a covariate, ΔΔQTcF as the dependent variable, and subject as a random effect for both intercept and slope, when applicable.

To account for the possible effects of the revefenacin metabolite THRX‐195518 on the estimation and prediction for ΔΔQTcF in the model, the following analysis was performed: the relationship between ΔΔQTcF and plasma concentrations of revefenacin and THRX‐195518 was investigated by a linear mixed‐effects modeling approach of the form: ΔΔQTcF*_ij_* = α*_i_* + β_1_ *_i_*(Conc*_ij_*) + β_2i_(Conc*_ij_) + ɛ*_ij_*, where Conc*_ij_* and Conc*_ij_ were concentrations of revefenacin and THRX‐195518, respectively, and the other notations were the same as those described above. The same 3 linear models as described above for revefenacin were investigated.

The best model was selected based on the following criteria: a significant reduction in the NONMEM objective function value (6.64 points; *P* < .01) and/or reduction in the Akaike information criterion value, and goodness‐of‐fit parameters/plots and physiologically reasonable and/or statistically significant estimates (95%CI does not include a zero) of mean parameters and their standard errors. From the final selected model, the population‐predicted ΔΔQTcF and its corresponding 2‐sided 95%CI were computed at the mean C_max_ (for the therapeutic and supratherapeutic doses) for revefenacin and THRX‐195518. Hysteresis in the relationship between ΔΔQTcF and plasma concentrations of revefenacin and THRX‐195518 was also investigated.

## Results

### Subjects

In total, 48 subjects (29 women and 19 men) enrolled in the study and received at least 1 dose of the study treatment (including placebo) and were included in the PK, pharmacodynamics (PD), and safety analysis sets. For PK/PD analysis, data from 42 subjects were used (6 subjects who did not receive a placebo dose were excluded from the analysis). Of the 48 subjects, 36 (19 women and 17 men) received all 4 study treatments and completed all study treatment periods.

Participants in the safety analysis set had a mean age of 34.7 years (range, 18–54 years) and a mean body weight of 72.0 kg (range, 57.3–92.7 kg).

### ECG Analysis

#### Effect on Heart Rate

**Figure 1:**
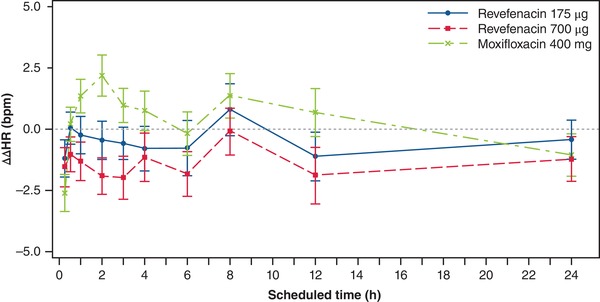
Effect of revefenacin on heart rate (HR). Mean ± SE placebo‐corrected change from baseline HR (ΔΔHR) across postdose times. Data points are shown at 0.25, 0.5, 1, 2, 3, 4, 6, 8, 12, and 24 hours postdose. bpm, beats per minute; h, hours; SE, standard error.

Inhaled revefenacin doses of 175 and 700 µg did not have a relevant effect on HR. Mean placebo‐corrected ΔHR (ΔΔHR) was within ±5 beats per minute at all postdose times (Figure 1).

#### Effect on Cardiac Repolarization: QT Interval

**Figure 2:**
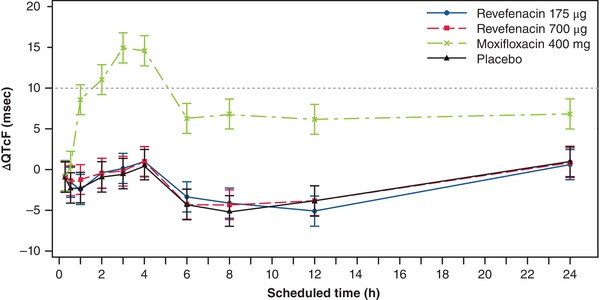
Effect of revefenacin on QTc. Least‐squares mean (±95%CI change) from baseline QTcF (ΔQTcF) by time and treatment. Data points are shown at 0.25, 0.5, 1, 2, 3, 4, 6, 8, 12, and 24 hours postdose. CI, confidence interval; h, hours; HR, heart rate; msec, milliseconds; QTcF, QT interval corrected for heart rate using Fridericia's formula.

Because revefenacin did not have an effect on HR exceeding 10 bpm, QTcF was used as the primary end point. Mean ΔQTcF was similar after administration of 175 and 700 µg revefenacin and placebo (Figure 2). The resulting ΔΔQTcF for revefenacin was therefore small at all postdose points. Following administration of revefenacin 175 and 700 µg, ΔΔQTcF values were close to 0 at all times, with the largest mean ΔΔQTcF of 1.0 millisecond (95%CI, −1.2 to 3.1 milliseconds) 8 hours postdose and 1.0 millisecond (95%CI, −1.1 to 3.1 milliseconds) 1 hour postdose after inhalation of revefenacin 175 and 700 µg, respectively. The upper bound of the 1‐sided 95%CIs was below 3.1 milliseconds at all times. QTc prolongation observed after dosing with moxifloxacin confirmed the study's assay sensitivity. Mean ΔΔQTcF for moxifloxacin was 10.8, 11.9, and 15.4 milliseconds at the 3 predefined times (1, 2, and 3 hours postdose, respectively) with all lower bounds of the 95%CI above 5 milliseconds (8.7, 9.8, and 13.3 milliseconds, respectively; Table 1). There were no QTcF outliers in terms of QTcF > 480 milliseconds or ΔΔQTcF > 60 milliseconds, and no T‐wave morphology changes were observed for any of the treatments (data not shown).

**Table 1:** Placebo‐Corrected Change From Baseline in QTcF (ΔΔQTcF) Across Postdose Times

	Least‐Squares Mean (95%CI), msec		
Time (h)	Moxifloxacin 400 mg	Revefenacin 175 µg	Revefenacin 700 µg
0.25	0.1 (−2 to 2.2)	0.2 (−2 to 2.3)	−0.0 (−2.1 to 2.1)
0.5	2.7 (0.6 to 4.8)	0.7 (−1.4 to 2.8)	0.9 (−1.2 to 3.0)
1.0	10.8 (8.7 to 12.9)	−0.2 (−2.3 to 1.9)	1.0 (−1.1 to 3.1)
2.0	11.9 (9.8 to 14)	0.4 (−1.7 to 2.5)	0.5 (−1.6 to 2.5)
3.0	15.4 (13.3 to 17.5)	0.6 (−1.5 to 2.7)	0.3 (−1.8 to 2.4)
4.0	14.0 (11.9 to 16.1)	0.4 (−1.8 to 2.5)	0.4 (−1.7 to 2.5)
6.0	10.5 (8.4 to 12.7)	0.9 (−1.2 to 3.1)	0.0 (−2.1 to 2.1)
8.0	11.9 (9.8 to 14)	1.0 (−1.2 to 3.1)	0.8 (−1.3 to 2.9)
12.0	10.0 (7.9 to 12.1)	−1.2 (−3.4 to 0.9)	0.0 (−2.1 to 2.1)
24.0	5.8 (3.7 to 7.9)	−0.4 (−2.5 to 1.7)	−0.1 (−2.2 to 2.0)

CI, confidence interval; h, hours; msec, milliseconds; QTcF, QT interval corrected for heart rate using the Fridericia's formula.

Two‐sided 95%CIs were used.

#### Effect on PR Interval and QRS Interval

Mean placebo‐corrected change from baseline PR was within ±3 milliseconds and mean placebo‐corrected change from baseline QRS was within ±1 millisecond for both doses of revefenacin (data not shown).

### Pharmacokinetics

**Figure 3:**
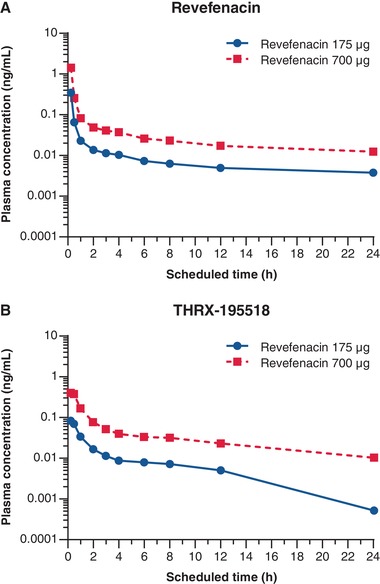
Plasma concentrations of (A) revefenacin and (B) THRX‐195518 across 24 hours following administration of inhaled single doses of revefenacin 175 and 700 µg. Data points are shown at 0.25, 0.5, 1, 2, 3, 4, 6, 8, 12, and 24 hours postdose. h, hours.

Plasma concentrations of revefenacin and THRX‐195518 were low after inhaled revefenacin administration, and peak concentrations were attained at the first sampling time (15 minutes postdose; Figure 3A and B, respectively). PK parameters for revefenacin and its primary metabolite (THRX‐195518) are shown in Table 2. Revefenacin exposure (based on AUC and C_max_) increased proportionally from 175 to 700 µg revefenacin; increasing the dose by 4‐fold resulted in an increase in mean revefenacin exposure of approximately 3.6‐ to 4.1‐fold. For THRX‐195518, a slightly greater than dose‐proportional increase in exposure was observed from 175 to 700 µg revefenacin; increasing the dose by 4‐fold resulted in an increase in THRX‐195518 exposure of approximately 5.0‐ to 5.5‐fold.

**Table 2:** Plasma Pharmacokinetic Parameters of Revefenacin and THRX‐195518 Following Administration of Inhaled Single Doses of Revefenacin 175 and 700 µg

	REVEFENACIN				THRX‐195518			
	REVEFENACIN 175 µg		REVEFENACIN 700 µg		REVEFENACIN 175 µg		REVEFENACIN 700 µg	
PK Parameter (Unit)	n		n		n		n	
AUC_24_ (ng·h/mL)^a^	40	0.28 (0.10)	44	1.02 (0.43)	NA	NR	41	0.98 (0.40)
AUC_last_ (ng·h/mL)^a^	43	0.27 (0.12)	45	0.99 (0.45)	40	0.17 (0.11)	44	0.93 (0.43)
C_max_ (ng/mL)^a^	43	0.35 (0.17)	45	1.42 (0.69)	40	0.09 (0.04)	44	0.42 (0.19)
T_max_ (h)^b^	43	0.25 (0.25, 0.33)	45	0.25 (0.25, 0.28)	40	0.25 (0.25, 0.52)	44	0.25 (0.25, 0.55)

AUC_24_, area under the plasma concentration‐time curve from time 0 to 24 hours postdose; AUC_last_, area under the plasma concentration time curve from time zero to the time of the last observed/measured nonzero concentration; C_max_, maximum observed plasma concentration; h, hours; n, number of observations; NA, not available; NR, not reportable; PK, pharmacokinetic; T_max_, time to reach C_max_.

a Presented as mean (standard deviation).

b Presented as median (minimum, maximum).

### C‐QTc Analysis

**Figure 4:**
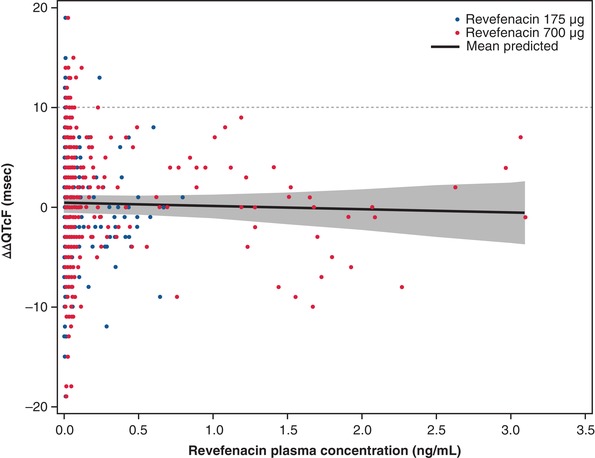
Model‐predicted ΔΔQTcF (mean and 90%CI) and observed ΔΔQTcF across deciles of revefenacin plasma concentrations. Blue and red circles denote the observed ΔΔQTcF for revefenacin 175 and 700 µg, respectively. The solid black line with gray‐shaded area denotes the model‐predicted mean ΔΔQTcF with 90%CI. ΔΔQTcF, placebo‐corrected change from baseline QT interval corrected for heart rate using Fridericia's formula; CI, confidence interval; msec, milliseconds.

**Figure 5:**
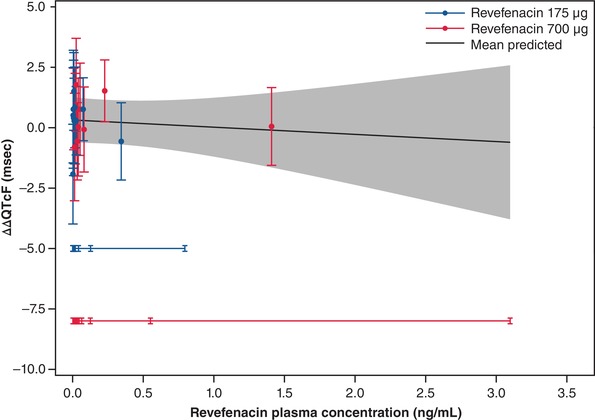
Model‐predicted ΔΔQTcF (mean and 95%CI) and observed ΔΔQTcF (mean and 95%CI) across deciles of revefenacin plasma concentrations. Blue and red circles with vertical bars denote the observed mean ΔΔQTcF with 95%CI displayed at the median plasma concentration within each decile for 175 and 700 µg revefenacin, respectively. The solid black line with gray‐shaded area denotes the model‐predicted mean ΔΔQTcF with 95%CI. The horizontal blue and red lines with notches show the range of concentrations divided into deciles for revefenacin 175 and 700 µg, respectively. ΔΔQTcF, placebo‐corrected change from baseline QT interval corrected for heart rate using Fridericia's formula; CI, confidence interval; msec, milliseconds.

By visual inspection of mean ΔΔQTcF and revefenacin concentration profile across times, it was concluded that hysteresis, a delay between mean C_max_ and the QT effect, was not present. A linear concentration‐QTc (C‐QTc) model with a fixed intercept was found to provide the best fit to the data and was therefore used to characterize the relationship between ΔΔQTcF and revefenacin (and THRX‐195518) plasma concentrations. Observed and model‐predicted ΔΔQTcF as a function of revefenacin concentration are shown in Figure 4. The goodness‐of‐fit plot, which shows the observed and predicted mean ΔΔQTcF (95%CI) within each revefenacin plasma concentration decile for doses of 175 and 700 µg, demonstrated that predicted ΔΔQTcF values were close to observed values (Figure 5). Using the model with revefenacin concentration as the covariate, the estimated slope of the C‐QTc relationship was −0.3 milliseconds per ng/mL (95%CI, −1.47 to 0.87 milliseconds per ng/mL), with a statistically nonsignificant intercept of 0.3 milliseconds (Figure 5). Using the model with both revefenacin and THRX‐195518 concentrations as the covariates, the estimated population slopes of the C‐QTc relationship was −1.5 milliseconds per ng/mL (95%CI, −3.10 to 0.06 milliseconds per ng/mL) for revefenacin and 4.8 milliseconds per ng/mL (95%CI, 0.68 to 8.92 milliseconds per ng/mL) for THRX‐195518. With both models, the predicted effect on ΔΔQTc was below 5 milliseconds across the observed range of revefenacin and THRX‐195518 plasma concentrations.

### Safety

Treatment‐emergent AEs (TEAEs) were reported in 3 of 42 subjects (7.1%) on placebo, in 6 of 43 subjects (14.0%) on revefenacin 175 µg, and 5 of 45 subjects (11.1%) on revefenacin 700 µg. The majority of TEAEs were mild in severity, and there were no severe or serious TEAEs or deaths. Headache (reported by 1 subject after receiving revefenacin 175 µg and 2 subjects after receiving revefenacin 700 µg) and dysgeusia (reported by 2 subjects after receiving revefenacin 175 µg and 1 subject after receiving revefenacin 700 µg) were the most frequently reported TEAEs after revefenacin administration. TEAEs considered possibly/probably related to revefenacin were reported by 4 subjects (9.3%) after administration of revefenacin 175 µg (glossodynia, nausea, oral paresthesia, dysgeusia, head discomfort, headache, and sensory disturbance) and by 5 subjects (11.1%) after administration of revefenacin 700 µg (tongue exfoliation, asthenia, fatigue, dysgeusia, headache, syncope, and hyperhidrosis). No cardiovascular TEAEs were reported.

## Discussion

The results of this study demonstrate that inhaled revefenacin at therapeutic (175‐µg) and supratherapeutic (700‐µg) doses and concentrations did not have a clinically meaningful effect on cardiac repolarization (the QTc interval) or cardiac conduction (PR and QRS intervals) in healthy subjects. The effect of both doses of revefenacin on mean ΔΔQTcF did not exceed 5 milliseconds, with the upper limit of all 2‐sided 95%CIs less than 10 milliseconds; therefore, this study represents a negative thorough QT study as defined by the ICH E14 guidance,^11^ and demonstrates that revefenacin will not cause QTc prolongation of clinical concern across therapeutic and supratherapeutic doses. The results were further substantiated by C‐QTc analysis, which demonstrated that a clinically relevant effect (ie, above 10 milliseconds) can be excluded within the observed range of revefenacin and THRX‐195518 plasma concentrations.

Abnormal prolongation of the QTc interval can increase cardiovascular mortality; in light of the high rates of cardiovascular comorbidities among patients with COPD, it is important that a drug used to manage COPD is not associated with negative cardiovascular effects.^12^, ^13^, ^14^ These patients have a 2 to 5 times elevated risk of ischemic heart disease, cardiac arrhythmia, and heart failure.^14^ Some case studies have reported LAMA and long‐acting beta agonist (LABA) treatments increase the risks of cardiovascular events in COPD patients^15^, ^16^; however, other studies, including prospective trials, have shown no elevated risks for cardiovascular events with LAMA or LAMA/LABA combinations.^17^, ^18^, ^19^, ^20^ Revefenacin has not been associated with adverse cardiovascular events in 2 identical double‐blind, placebo‐controlled, 12‐week phase 3 trials (NCT02512510, NCT02459080)^21^ or in a 52‐week double‐blind phase 3 study (NCT02518139)^22^ of patients with moderate to very severe COPD.

Although this thorough QT study was conducted in healthy volunteers, it further demonstrates absence of a clinically relevant effect of revefenacin on ECG parameters at exposures in excess of those associated with a therapeutic revefenacin dose. The absence of a relevant effect is also consistent with the weak activity of both revefenacin and THRX‐195518 at the hERG channel.

Use of an unblinded active control (moxifloxacin) in this study was in accordance with findings from the FDA IRT, demonstrating that assay sensitivity (similar QTc effect) could be achieved with either open‐label or blinded moxifloxacin.^23^ This same recommendation was also made in the Q&A document by the ICH E14 Implementation Working Group (Question 3 [ICH E14 Q&A, 2015]).^24^

The PK results obtained in this study are consistent with those of previous studies in COPD patients.^6^, ^7^ Inhaled revefenacin and its primary metabolite, THRX‐195518, appeared rapidly in the systemic circulation in healthy subjects, with peak concentrations achieved at the first PK sampling time, 15 minutes after onset of nebulization. Plasma concentrations of revefenacin and THRX‐195518 were low and decreased in a biexponential fashion after reaching C_max_. The overall and maximum exposures increased proportionally for revefenacin and slightly more than dose proportionally for THRX‐195518 with increasing revefenacin dose from 175 to 700 µg.

Revefenacin administered at both the therapeutic (175‐µg) and the supratherapeutic (700‐µg) doses appeared to be well tolerated by the participants in this study. The majority of TEAEs were mild in severity; there were no severe or serious TEAEs or deaths. This is consistent with the safety profile for revefenacin observed in previous studies: administration of once‐daily revefenacin 88 and 175 µg through a standard jet nebulizer was well tolerated and produced clinically and statistically significant improvements in trough forced expiratory volume in 1 second relative to placebo at 12 weeks in 2 identical phase 3 trials of patients with moderate to very severe COPD.^21^

A potential limitation of this study is that it did not include patients with COPD who have a higher exposure to the metabolite THRX‐195518 than healthy subjects.^6^, ^7^ Metabolite levels observed in the healthy subjects receiving the 700‐µg revefenacin dose in this study were, however, in excess of those reported in patients with COPD at the therapeutic dose (175 µg) and thus represent a higher exposure than anticipated in clinical use. Another potential limitation is that only a single dose was tested; however, accumulation of both revefenacin and THRX‐195518 is minimal after repeat daily dosing, with similar C_max_ values observed after single and repeat dosing.^7^ Therefore, the findings from this study satisfy the recommendation that QT interval prolongation be tested at exposures in excess of the therapeutic range.

## Conclusions

Inhaled revefenacin at doses of 175 and 700 µg did not have a clinically meaningful effect on cardiac repolarization (QTcF) or cardiac conduction (PR and QRS interval) and was generally well tolerated by the participants. Overall, these results represent a negative thorough QT study consistent with ICH E14 criteria and support the observation that revefenacin does not pose adverse cardiovascular risks and can be safely administered to patients with COPD.

## Conflicts of Interest

Marie T. Borin is a consultant for and received financial support from Theravance Biopharma US, Inc. Borje Darpo is a consultant for and owns stock in ERT. Hongqi Xue is an employee of ERT. David L. Bourdet is an employee of Theravance Biopharma US, Inc. Chris N. Barnes and Srikanth Pendyala were employees of Theravance Biopharma US, Inc. at the time this study was conducted.

## Funding

This study was funded by Theravance Biopharma, Ireland Limited Inc. (Dublin, Ireland).

## Data‐Sharing Statement

Theravance Biopharma (and its affiliates) will not be sharing individual deidentified participant data or other relevant study documents.
